# Up, down, and all around the epigenomic regulation of metabolism

**DOI:** 10.1186/1753-6561-6-S3-O14

**Published:** 2012-06-01

**Authors:** Mitchell A Lazar

**Affiliations:** 1 Division of Endocrinology, Diabetes, and Metabolism, and The Institute for Diabetes, Obesity, and Metabolism, Perelman School of Medicine and the University of Pennsylvania, Philadelphia, PA 19104 USA

## 

Nuclear receptors (NRs) transduce environmental and metabolic signals into alterations in gene expression by recruiting coregulators that alter chromatin structure. Rev-erbs alpha and beta (NR1D1 and NR1D2, respectively) are heme receptors that comprise negative limb of the circadian clock. The Rev-erbs function in the genome by recruiting complexes containing the nuclear receptor corepressor NCoR and histone deacetylase 3 (HDAC3). The histone modifying enzymatic activity of HDAC3 depends upon interaction with NCoR or SMRT, and disruption of this corepressor function alters circadian rhythms and metabolic physiology. In liver, circadian expression of Rev-erbs leads to their oscillating interaction with the genome. At specific times of day the Rev-erbs recruit NCoR and HDAC3 to thousand of genomic sites, with an enrichment for genes regulating lipid metabolism, and with a rhythm that is anti-phase to histone acetylation at these sites. The cistromes of Rev-erbs alpha and beta are nearly identical, and the coordinated activities of the Rev-erbs protect the clock and normal metabolic function. Thus Rev-erbs, NR corepressors, and HDAC3 team up to orchestrate a circadian epigenomic rhythm that plays a major role in the regulation of intermediary metabolism and nutrient storage in the liver. This pathway links the circadian environment to the epigenome, resulting in a daily re-routing of metabolites into lipid versus glucose metabolic pathways that is required for the normal physiological control of metabolism.

